# Stereoscopic Near-Infrared Fluorescence Imaging: A Proof of Concept Toward Real-Time Depth Perception in Surgical Robotics

**DOI:** 10.3389/frobt.2019.00066

**Published:** 2019-08-16

**Authors:** Maxwell J. Munford, Ferdinando Rodriguez y Baena, Stuart Bowyer

**Affiliations:** ^1^Department of Mechanical Engineering, Imperial College London, London, United Kingdom; ^2^Department of Electrical and Electronic Engineering, Imperial College London, London, United Kingdom

**Keywords:** active constraints, stereoscopic near-infrared fluorescence, depth perception, real-time image acquisition, NIRF imaging

## Abstract

The increasing use of surgical robotics has provoked the necessity for new medical imaging methods. Many assistive surgical robotic systems influence the surgeon's movements based on a model of constraints and boundaries driven by anatomy. This study aims to demonstrate that Near-Infrared Fluorescence (NIRF) imaging could be applied in surgical applications to provide subsurface mapping of capillaries beneath soft tissue as a method for imaging active constraints. The manufacture of a system for imaging in the near-infrared wavelength range is presented, followed by a description of computational methods for stereo-post-processing and data acquisition and testing used to demonstrate that the proposed methods are viable. The results demonstrate that it is possible to use NIRF for the imaging of a capillary submersed up to 11 mm below a soft tissue phantom, over a range of angles from 0° through 45°. Phantom depth has been measured to an accuracy of ±3 mm and phantom angle to a constant accuracy of ±1.6°. These findings suggest that NIRF could be used for the next generation of medical imaging in surgical robotics and provide a basis for future research into real-time depth perception in the mapping of active constraints.

## Introduction

Many assistive surgical robotic systems influence the surgeon's movements based on a model of constraints and boundaries driven by anatomy. Models are generated of the critical soft tissue regions which cannot be pierced and these are used to guide the surgeon in a safe and cooperative manner. The development of Near-Infrared Fluorescence (NIRF) imaging technology presents a possibility for the subsurface stereoscopic imaging of soft tissues. Within the context of active constraints, NIRF could be applied for 3D real-time imaging of anatomical features beneath the tissue surface.

Monoscopic fluorescence imaging is a widely researched technology for the real-time assessment of phantom tissues of interest (Schaafsma et al., 2011), with large volumes of research focusing on optimizing dose for image quality (Hutteman et al., 2012). Existing literature suggests that the feasibility of Indocyanine green (ICG) fluorescence imaging has already been validated in specific applications (Schaafsma et al., 2011; Hutteman et al., 2012). These are currently limited to the 2 dimensional mapping of small capillary networks, such as lymph nodes and liver metastases whereby concentrated ICG is injected into the area of interest, excited, and then imaged (van der Vorst et al., 2011).

**Figure 1:**
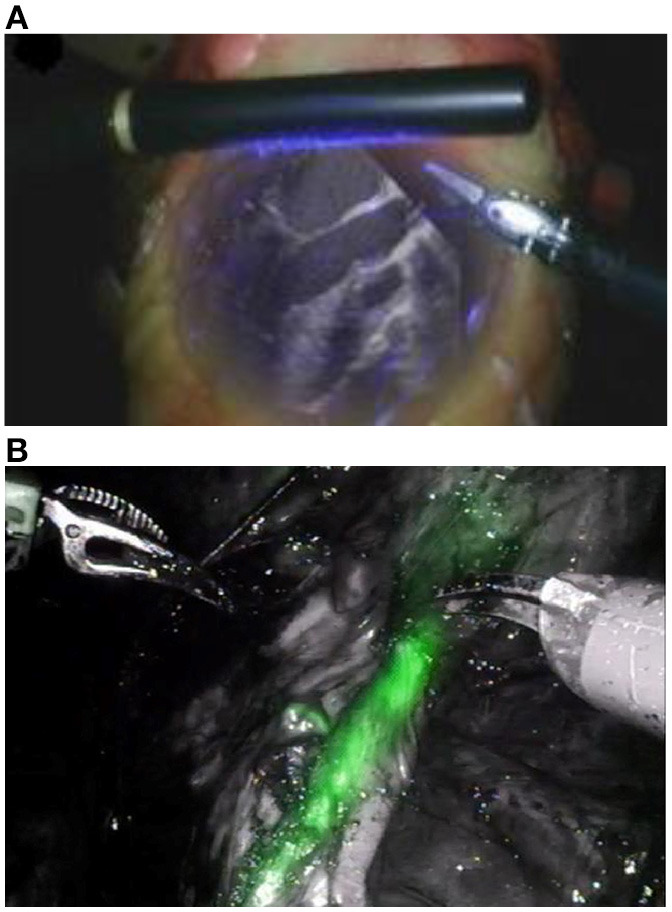
**(A)** Augmented visible light and ultrasound image from Intuitive Surgical's Da Vinci ultrasound probe (Lee et al., 2010) and **(B)** visible light overlaid with NIRF from the Pinpoint Novadaq system (Kaplan-Marans et al., 2019). Both images are taken from real time video. Reproduced with permission from Elsevier.

Figures 1A,B illustrate how the application of combined visible and non-visible light imaging can be applicable to achieve subsurface imaging during surgery. In Figures 1A,B, respectively, the Da Vinci system utilizes an ultrasound probe while the Novadaq system employs monoscopic fluorescence imaging of injected ICG to image subsurface tissues (Medizintechnik, 2016). Despite the Novadaq system being monoscopic it produces a notably greater resolution image than Da Vinci's ultrasound probe.

The Pinpoint system, from Novadaq, in Figure 1B is an example of an existing system utilizing monoscopic fluorescence imaging (Lee et al., 2010; Medizintechnik, 2016). In Figure 1B, ICG has been injected into soft tissues then excited and imaged, shown in green. Both imaging systems provide a 2D image of similar resolution, however, boundaries between the marked and unmarked tissue in the NIRF augmented image (Figure 1B) are equally sharp over the whole field of view. In contrast, the subsurface tissues of interest in the ultrasound image (Figure 1A) shown in white are less clear at the periphery of the viewing region making it difficult for a surgeon to infer tissue locations. Furthermore, light leakage can affect image quality in ultrasound augmented images, as indicated by the purple light in Figure 1A, as light in some wavelength ranges may fail to be removed by filtering. This highlights some benefits of near-infrared fluorescence over ultrasound.

Success in high resolution and real-time monoscopic NIRF imaging provides a basis to believe that stereoscopic NIRF could be used to identify active constraints in surgical applications. It is thought that a two camera stereo probe system could be used, initially in open surgery.

Technologies currently exist in robotic and adaptive surgical devices to prevent a surgeon from passing certain boundaries (Bowyer and Rodriguez y Baena, 2014, 2015), however, there are no methods in place to monitor moving (active) constraints, which are common in cardiac and lung surgery. Collaborative, in contrast to autonomous, control systems are the preferred technology in the majority of commercially available surgical robotic products (Bowyer and Rodriguez y Baena, 2013). In such systems, the accurate evaluation of active constraints is critical to the robot functionality. Some attempts have been made to model dynamic constraints pre-operatively, however, these have been unsuccessful due to the number of variables required to form an accurate model (Kwok, 2012).

This study aims to demonstrate that NIRF imaging could be applied in surgical applications to provide subsurface mapping of capillaries beneath soft tissue. This study focuses on the use of NIRF to determine submersion depth and inclination angle of a phantom below a soft tissue phantom.

## Materials and Methods

**Figure 2:**
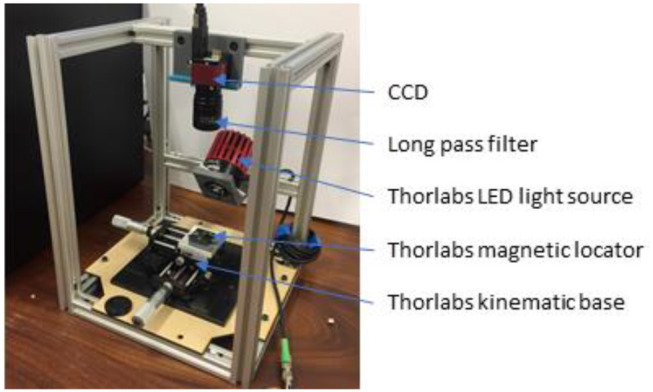
Assembled test rig used for ICG excitation, specimen positioning and data collection.

All testing was carried out using the custom-built test rig shown in Figure 2. It consists of an excitation LED, a 2-degree-of-freedom translation linear stage and kinematic base, a modified Charge-Coupled Device (CCD) camera (Allied Vision Guppy Pro F-146 Color) and long pass filter (MIDOPT LP780). The camera was modified to remove its infrared (IR) cut filter to allow image capture in the IR spectrum and an additional filter fitted to remove visible light.

The excitation LED was a 800 mW source of 780 nm light, chosen to emit on the peak excitation of ICG pigment. ICG pigment was used in solution for fluorescence as it is FDA approved and well-studied (Starosolski et al., 2017).

**Figure 3:**
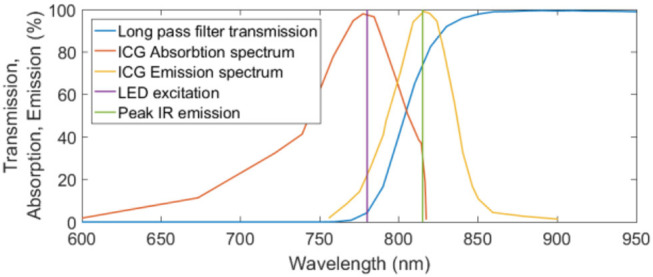
Transmission, absorption, and emission spectra for the MIDOPT filter, ICG excitation, and ICG emission respectively. Peak IR excitation and the wavelength of the excitation LED are also shown.

The excitation light source was a Thorlabs M780LP1 Mounted LED, with a heat sink operated under current control to ensure safe function. It is noted that this LED had a gaussian emission between 700 and 825 nm, not the peak emission as in Figure 3. There may have been some overlap between the LED light and the cutoff filter, however, this was deemed acceptable as this wavelength light lies within the absorption spectrum of ICG (Thorlabs, 2019).

Application of Wien's law and the Stefan-Boltzmann law to incident radiation on skin suggested that an 800 mW source of 780 nm light would cause a maximum temperature increase of 10°C at steady state (Incropera et al., 2011). This could cause damage to sensitive tissues, such as the eyes so was shielded during operation.

The linear stage and kinematic base allowed for precise and reliable control of the specimen position, an important factor in stereoscopic calibration. Imaging proceeded in this study with only one camera. The specimen was translated 0.6 mm between left and right image capture to simulate the separation distance of a stereo pair of cameras. This set up allowed for a reliable, high resolution positional placement of the test specimen with translation accuracy of 10 μm in the X and Y directions via adjustment of the micrometer heads. Dimensions of the field of view dictated by this camera set up, with a distance of 130 mm between the CCD sensor and object plane, are given in Table 1.

**Table 1:** Dimensions of the CCD and object planes and the resulting field of view (FOV).

**Field of view**			
Image	Vertical	1,038	pixels
		4.83	mm
	Horizontal	1,388	pixels
		6.45	mm
Object	Vertical	21.02	mm
	Horizontal	28.11	mm
	Resolution	50	Pixel/mm
FOV	Vertical	20.37	mm
	Horizontal	28.11	mm

Connection of the camera to a computer was made using a firewire PCI interface, which allowed image acquisition, and subsequent processing, to be done using MATLAB R2018a (Mathworks inc.).

### Stereoscopic Image Post-processing

**Figure 4:**
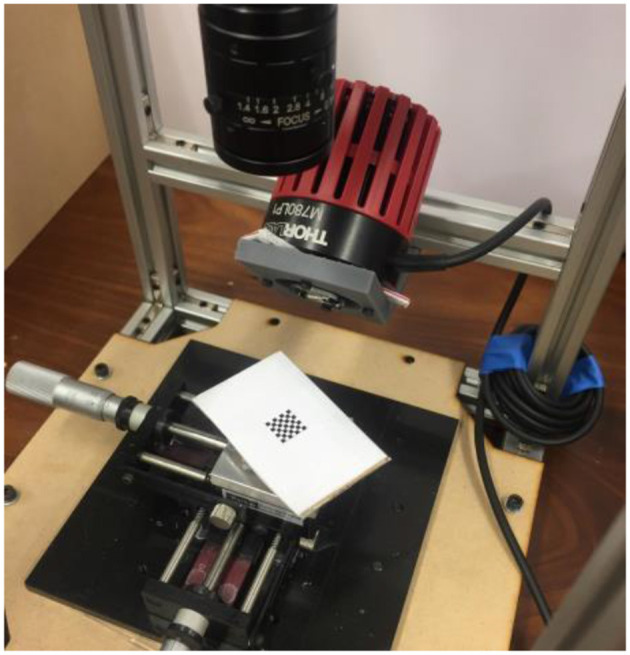
Example of the set up used for calibrating the camera stereo parameters. The calibration squares were used in a number of orientations and positions.

Stereo imaging requires a calibration process that determines the extrinsic parameters, which describe the scene layout, of the camera. This was done by capturing a series of stereo image pairs of chequerboard squares, as shown in Figure 4, and calculating a mapping from real world square size to image square size.

Disparity maps were produced based on an algorithm which searched for pixels in the right image which were corresponding pixels to each pixel in the left image. This process implemented a scanning algorithm and a cost function to assign corresponding pixels.

Invertibility between these the disparity maps was also checked as this is a requirement for consistent stereo mapping (Chang et al., 2017). The scanning algorithm iterated through each pixel position *(i,j)* in the left image and searched the range of pixels *(i-WinRad* < *i* < *i* + *WinRad, j)* in the right image for correspondence. A pixel window radius of *WinRad* = *9 pixels* was used, this dictated the size of the region of pixels which were searched for correspondence.

**Figure 5:**
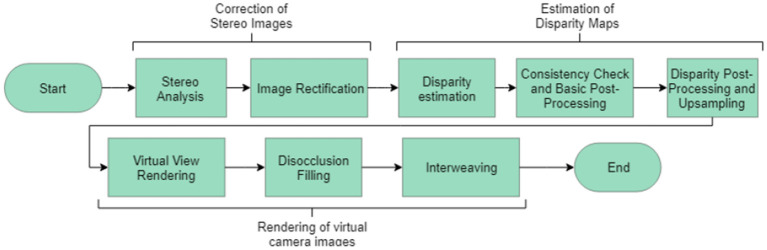
Overview of the algorithm implemented to generate depth maps and reconstruct scenes from stereo images (Haralick and Shapiro, 1992).

When evaluating pixels for correspondence, a pixel was assigned as corresponding if it minimized the chosen cost function. Two choices of cost functions were implemented and their resulting depth maps compared to ground truth known values. Comparison of a wider variety of cost functions was outside the scope of this study. The choices of cost function were a simple pixel intensity measure, Equation 1, and a normalized cross correlation measure, Equation 2 (Gedam et al., 2017). Equation 2 shows the cost function utilized in the algorithm shown in Figure 5. Both cost functions shown here were implemented from works by Haralick and Shapiro (1992) and Lewis (1995).

(1) $$\begin{array}{r} C_{lr}=|{I\left(i,j\right)}_{l}-\;{I\left(k,j\right)}_{r}| \end{array}$$

(2) $$\begin{array}{r} C_{lr}=\;\frac{\left|\underset{WinRad}{\sum }\left(I_{l}\left(i,j\right)-\;\bar{I_{l}}\right)\;\times \;\left(I_{r}\left(k,j\right)-\;\bar{I_{r}}\right)\right|}{\sqrt{{\underset{WinRad}{\sum }\left(I_{l}\left(i,j\right)-\;\bar{I_{l}}\right)\;}^{2}\times \;{\underset{WinRad}{\sum }\left(I_{r}\left(k,j\right)-\;\bar{I_{r}}\right)\;}^{2}}}\; \end{array}$$

Where *C*_*lr*_ is the assigned cost which is sought to be minimized, *I*(*i, j*)_*l*_ is the intensity value of pixel (*i, j*) in the left image, *I_l_* is the mean intensity across the chosen window radius and $\sum_{WinRad}\left(I_{l}\left(i,j\right)-\;I_{l}\right)$ represents the sum of deviations of each pixel from the mean intensity across the window radius.

Equation 2 yielded a more accurate pixel disparity and depth value than Equation 1, however, it was more computationally expensive with a run time of 20.4 s compared to 4.6 s for the cost function in Equation 1 (Windows 10, i7, HP EliteDesk 800 G3 TWR).

The cost function shown in Equation 2 provided an absolute value of the cost, standardized with respect to the mean intensity of each image. Some sources suggest that it is possible to implement analysis to locate important edges in the capture images (Gedam et al., 2017). This would naturally improve the quality of the image, however, these algorithms were not included here. Implementation of the scanning process and the cost function contributed to the overall algorithm which had the structure shown in Figure 5.

Depth maps of the scene were created by locating corresponding pixels, and their pixel disparity, in the stereoscopic image pairs, checking for consistency, and mapping from pixel disparity to real world distance via the mapping formed during calibration.

A parameter study showed that the quality of produced depth map varied with the window radius used to determine pixel disparity. Similarly, window radius was shown to affect the percentage of pixels for which no corresponding pair can be found, computation time, and depth map resolution.

Preliminary work suggested that glass capillaries of 4 mm internal diameter filled with ICG pigment in solution at 1 mg/ml could be used for proof-of-concept purposes. Capillaries were held in position in a 3D printed test piece filled with a milk and gelatin mixture that replicates the optical properties of soft tissue (Haralick and Shapiro, 1992). The choices made for the concentration of ICG, capillary diameter and soft tissue substitute are all supported by existing literature (Waterworth et al., 1995; Frangioni, 2003).

A calibration procedure was run before each series of testing, which formed a model of the stereo extrinsic parameters. This allowed for depth reconstruction of the subsurface scene based on pixel disparities between the left and right images. Once in position, the capillary specimens were excited at 780 nm and the resulting fluorescence, with peak emission at 815 nm, was captured in stereo. During testing, the whole rig was shielded from ambient light. Depth maps of the field of view were then generated via post-processing in MATLAB. Stereo post-processing algorithms were written to implement a “winner-takes-all” strategy and determine corresponding pixels between images based on the normalized cross correlation cost function in Equation 2. Preliminary work identified that an optimal window radius that maximized correspondences could be found, however, this was not implemented in this study.

**Figure 6:**
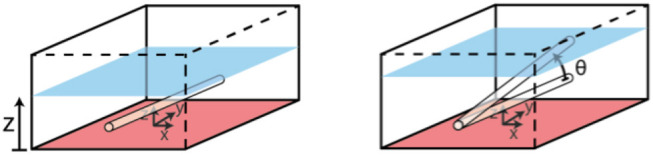
Set up for testing of phantom submersion depth and incline angle. Controlled variables are z and ϑ.

Testing sought to determine the effect of capillary submersion depth and inclination angle, as per the set up in Figure 6, on the imaging quality.

Depth maps were generated from the raw stereoscopic image data, and the number of corresponding pixels was found as a percentage of the number of pixels in the FOV (x by y). Absolute mean errors of the smoothed raw data against the ground truth geometry was also found, where ground truth geometry was calculated analytically, as the height of a cylindrical capillary at the given submersion depth and incline angle.

## Results

**Figure 7:**
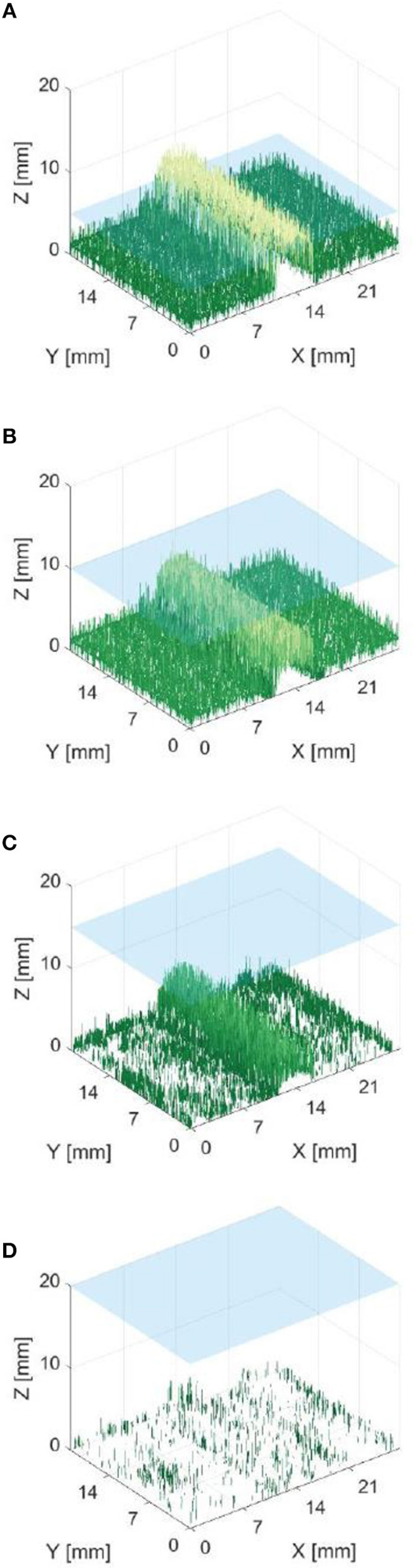
Reconstructed depth maps for a capillary specimen submerged beneath 1, 6, 11, and 16 mm **(A–D)**. Phantom surface shown in blue.

Figure 7 shows that it was possible to capture images of the capillaries fluorescing in the near-infra-red region of light. Figures 7A–D provide depth maps of the 21 mm by 28 mm field of view and demonstrate that the test rig and methods for data capture and post-processing described previously could be used to provide stereo images and an estimate of depth perception, to within ±3 mm.

The resulting depth maps for the case of a horizontally orientated capillary over a variety of capillary submersion depths are shown in Figures 7A–D. The blue surface indicates the top of the phantom and the z axis for height includes the 4 mm capillary tube. Comparing these figures suggests that this method of imaging is less capable of locating features which are deeper in the solution. This is as would be expected because both the excitation and emission light have a limited penetration depth. This depth is likely to be dependent on the tissue material and geometry and light intensity and power. Material properties including scattering and absorption also affect this.

**Figure 8:**
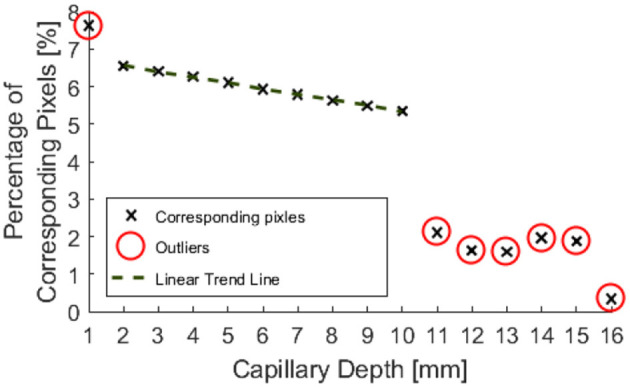
Percentage of pixels for which a corresponding pixel could be found in the partnering stereo image, for controlled submersion depth.

Figure 8 indicates that data from phantom submersion depths below 11 mm exhibited a distinct reduction in the number of corresponding pixels which were found. These data points were marked as outliers and excluded from further calculation of errors. This was further supported by data collected for phantom inclination angle, where it was observed that larger angles give a reduction in the percentage of corresponding pixels. This was most likely because a greater number of points are below 11 mm for a larger specimen angle.

**Figure 9:**
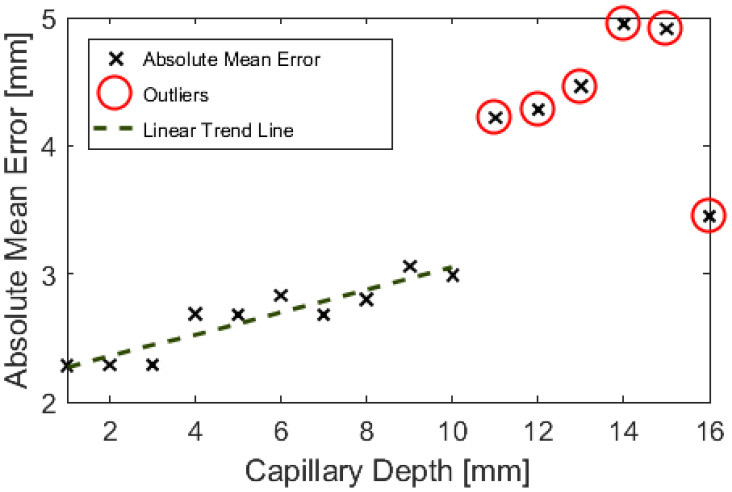
Absolute mean error in depth estimation of the entire field of view against controlled submersion depth.

Outliers in the resulting data are highlighted in Figures 8, 9, **11**, **12** and were excluded from calculation of trend lines.

Outliers in Figure 8 further support the hypothesis that the maximum imageable submersion depth was 11 mm as data points beyond this depth were spurious and noisy. Figure 9 suggests that the error in depth measurement was linear, ranging from ±2.3 to ±3 mm. Absolute errors can be expected to increase with capillary submersion depth as pixel errors imply a larger absolute error at greater submersion depth. These values are large compared to the data, however, this may be explained by both by the inherent error in adjusting the camera separation distance between left and right images and the limitations of the relatively simple algorithms used to allocate corresponding pixels. It is possible that the normalized cross product algorithm implemented as per Equation 2 incorrectly allocated corresponding pixels, causing inconsistencies in the disparity and there some level of error and noise in the data.

**Figure 10:**
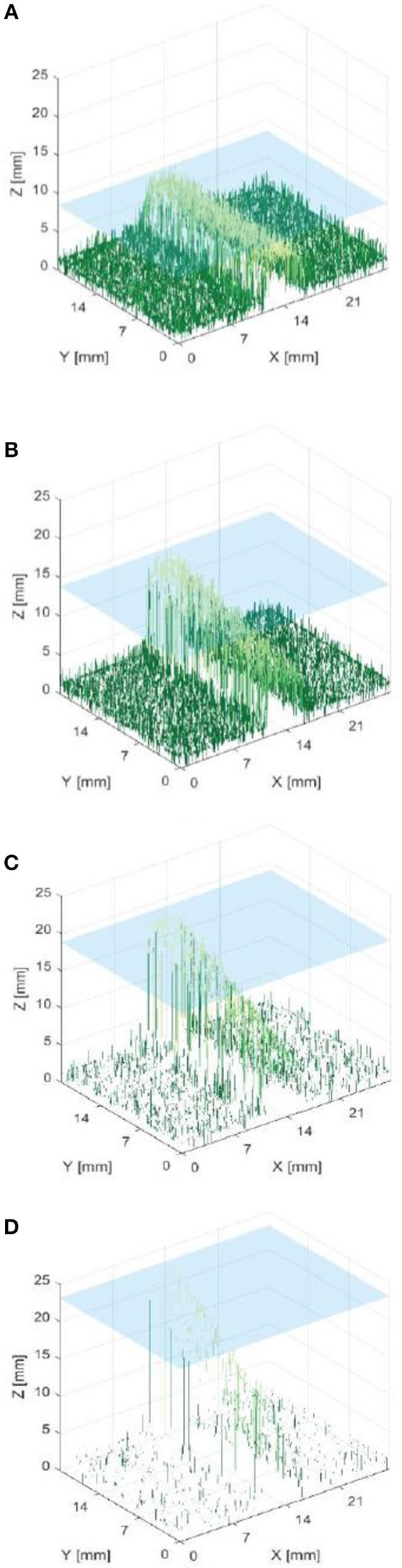
Reconstructed depth maps for a capillary specimen positioned at various angles: 0, 15, 30, and 45° **(A–D)**. Phantom surface shown in blue.

Figure 10 indicates that the ability to image the surface of a test specimen decreases as the incline angle increases. One aspect of this test which limits the validity of conclusions which can be drawn, is the fact that the depth of the specimen changes along its length. Given the previous trends suggested in Figures 8, 9, it is known that a change in depth will affect the quality of depth map which can be formed from stereo images of the specimen.

**Figure 11:**
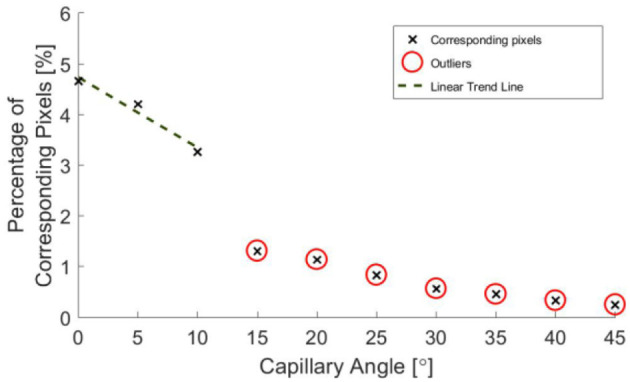
Percentage of pixels for which a corresponding pixel could be found in the partnering stereo image, for controlled incline angles.

Post-processing allowed for the inclination angle to be accurately inferred from the depth map data to within ±1.6°, as shown in Figure 11.

**Figure 12:**
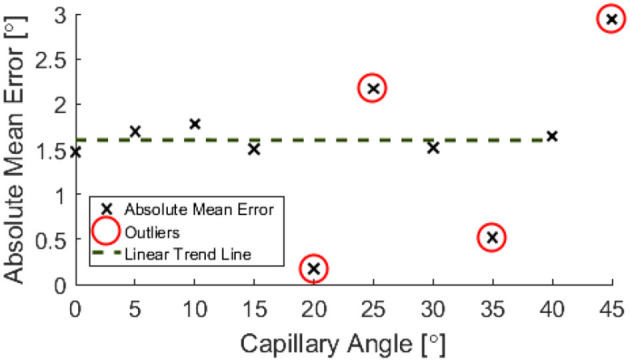
Absolute mean error in phantom angle against controlled angle.

A decrease in the number of corresponding pixels is likely to be a direct result of the penetration depth of incident light being less than the specimen depth. This occurs as the algorithms are less able to find corresponding pixels where the image appears more uniform. Figure 11 suggests that a linear trend is still present for greater angles and there may be a wider trend present if the depth was held constant. It is, however, not possible to draw conclusions from this region of the data without further experimentation, as the depth varies over the range of the specimen.

When calculating an estimate for test specimen angle, all data points below 11 mm were neglected. The test specimen angle was approximated by considering the geometry of the central range of columns of the depth map and taking a mean of these angle approximations.

**Figure 13:**
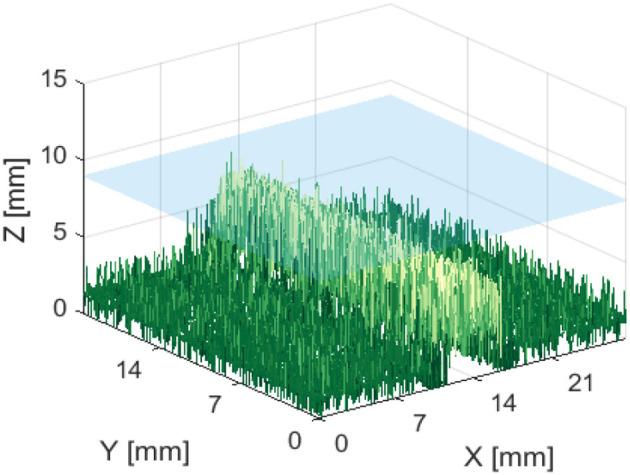
Reconstructed depth map for a capillary phantom 5 mm below the surface of soft tissue emulate, shown in blue.

**Figure 14:**
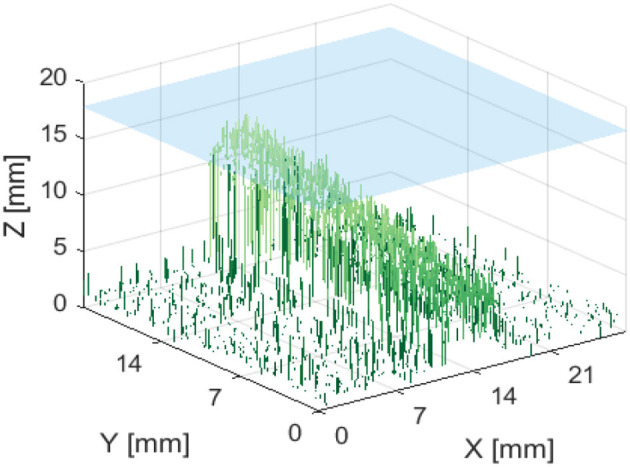
Reconstructed depth map for a capillary phantom inclined at 15° to the plane. Positioned so that the maximum height is 2 mm below the surface of soft tissue emulate, shown in blue.

Figures 13, 14 are examples of the reconstructed depth maps that were produced using NIRF imaging with a subsurface capillary. The data is representative of the original surface, despite being noisy.

## Discussion and Conclusion

It has been shown that it is possible to use NIRF for the imaging of a capillary submersed up to 11 mm below a soft tissue phantom, over a range of angles from 0 to 45°. Phantom depth has been measured to a worse case accuracy of ±3 mm and phantom angle to a constant accuracy of ±1.6°. The errors associated with the test data are within what is generally considered acceptable in a prototyped clinical scenario. It is thought that the constant error in reconstructed phantom angle could be due to the refractive index mismatch of the air-soft tissue interface causing a change in speed of the light. This is supported by similar results seen in light microscopy with penetration depth and resolution (Boothe et al., 2017; Stocker et al., 2017).

Algorithms were developed to form depth maps of a scene given a stereo image pair. Some study of the parameters which govern how well these stereo algorithms function was also completed. Images were also captured via NIRF, demonstrating that the combination of modified camera, filter and excitation light was suitable.

As shown in Figures 7–14, stereoscopic image processing was successfully applied to determine the submersion depth and incline angle of a fluorescent test specimen. With these results, it was possible to demonstrate that an ICG specimen can be imaged in stereo under NIRF imaging in conditions that could be mapped onto those seen in medical applications. This is an exciting early finding toward the incorporation of dynamic active constraints to protect deep-seated features within tissue.

Possible future areas of research will revolve around the use of 2 cameras for real-time imaging, a flow rate of pigment in the capillary and augmented reality to overlay active constraints on to captured video. Additionally, the acceleration of image processing algorithm would be required for real-time imaging and that the use of a designated IR camera sensor and more uniform distribution of excitation light could improve image quality. This would reduce the sensitivity of image results to IR light attenuation.

Furthermore, the LED used had an emission spectrum which is likely to have caused significant excitation light leakage and a high noise floor. In future studies this should be improved by implementing a long pass filter to remove such effects. This would provide improved images and a reduced noise floor.

It is also acknowledged that the methods here could be improved by measuring any temperature increase in the specimen to monitor any tissue damage caused by the power density of excitation light.

These findings suggest that NIRF could indeed be used for the next generation of medical imaging in surgical robotics and provide a basis for future research into real-time depth perception.

## Author Contributions

All work has been carried out by MM under the supervision of FR. Initial ideas for this proof of concept came from SB.

### Conflict of Interest Statement

The authors declare that the research was conducted in the absence of any commercial or financial relationships that could be construed as a potential conflict of interest.
